# The contribution of washing processes of synthetic clothes to microplastic pollution

**DOI:** 10.1038/s41598-019-43023-x

**Published:** 2019-04-29

**Authors:** Francesca De Falco, Emilia Di Pace, Mariacristina Cocca, Maurizio Avella

**Affiliations:** Institute for Polymers, Composites and Biomaterials, National Research Council of Italy, Via Campi Flegrei, 34 - 80078 Pozzuoli, NA Italy

**Keywords:** Characterization and analytical techniques, Environmental monitoring

## Abstract

Microplastic pollution caused by washing processes of synthetic textiles has recently been assessed as the main source of primary microplastics in the oceans. Therefore, understanding the effective contribution of the washing process of synthetic clothes to this environmental problem, is of great importance. In this study, wash trials at real scale were performed on commercial clothes by using a household washing machine in order to gain reliable data about the release of microplastics, and to identify possible influences of textile characteristics on the release. The wastewater was collected and filtered through subsequent filters with decreasing porosity, and the amount and dimensions of microfibres were determined. Microfibre release was analysed in relation to the nature and characteristics of the washed clothes. Results showed that microfibres released during washing range from 124 to 308 mg for kg of washed fabric depending from the type of washed garment that corresponds to a number of microfibres ranging from 640,000 to 1,500,000. Some textile characteristics, such as the type of fibres constituting the yarns and their twist, influenced the release of microfibres during washing. A great amount of microfibres of cellulosic nature was also released during washing of clothes made with a blend of polyester/cellulose. Finally the most abundant fraction of microfibres shed was retained by filters with pore size of 60 µm, presenting an average length of 360–660 μm and an average diameter of 12–16 μm, indicating dimensions that could pass through wastewater treatment plants and pose a threat for marine organisms.

## Introduction

Microplastic pollution caused by washing processes of synthetic textiles was discovered to be one of the main source of primary microplastics^1^. First accounts of synthetic fibres coming from clothes-washing machines were found in sludge, sludge products, and sewage treatment plant effluents^2,3^; moreover, as microplastics they were detected in different samples collected from beaches and from estuarine and subtidal sediments in the UK^4^. Nevertheless, the first study that clearly pointed out how the washing of synthetic clothes could be responsible for marine microplastic pollution, was the one of Browne *et al*.^5^. Through a forensic evaluation of microplastics from sediments collected on worldwide beaches (i.e. Australia, Oman, Chile, Philippines, Portugal, USA, Mozambique, UK, etc.), they discovered that the proportions of polyester and acrylic fibres used in clothing are similar to those found in habitats that receive sewage-discharges and sewage-effluent itself.

The release of microplastics from synthetic clothes is mainly caused by the mechanical and chemical stresses that fabrics undergo during a washing process in a laundry machine, which lead to the detachment of microfibres from the yarns that constitute the textile. Due to their dimensions, the released microfibres could partially pass through wastewater treatment plants (WWTPs) and reach directly the oceans. Actually, it is an open debate if such microfibres can, and in what proportion, be blocked by WWTPs. A great abundance of microfibres was found in effluents from 8 WWTPs in San Francisco Bay in California, USA^6^. Other studies detected the presence of microplastics in the effluents of WWTPs in Sweden^7^, in Australia^8^ and in Finland^9^, regardless if the WWTP had high efficiency or advanced treatment processes. As concluded by Talvitie *et al*.^9^, even if low concentrations of microplastics can pass through WWTPs, considering the large volumes of effluents discharged to the aquatic environment, eventually WWTPs have the potential to act as a pathway to release microplastics. In fact, the occurrence of microplastics in marine ecosystems is well documented by several works. A recent review related to microfibres detection in real samples^10^ highlighted how microfibres can be found in beaches worldwide, in the water of the Pacific Ocean, the North Sea, the Atlantic Ocean and even in the Artic and in deep sea sediments. Textile fibres were also found in fish and shellfish on sale for human consumption, sampled from markets in Makassar, Indonesia, and from California, USA^11^. Regarding possible effects on marine fauna, it was reported that polyethylene terephthalate (PET) microfibres ingested by the zooplankton crustacean Daphnia magna, could cause an increased mortality of the species^12^.

Recent estimations^1^ have assessed that synthetic clothes contributes by about 35% to the global release of primary microplastics to the world oceans, thus becoming the main source of microplastics. This estimation is not surprising considering that synthetic fibres represent almost the 60% of the annual global consumption of fibres, that is 69.7 Mt, used in the apparel industry^1^ and that, globally, more than 840 million domestic washing machines are used, consuming annually around 20 km^3^ of water and 100 TWh of energy^10^. In such scenario, it is of striking importance to evaluate the real environmental impact of washing processes of synthetic clothes, starting from quantifying microplastics that can be released during a wash and identifying possible parameters of influence on the release. Several works have already been published on this topic but, since all of them used different methodologies, clear comparisons are difficult. Most of these works performed washing tests in washing machines^13–16^ while others carried out laboratory simulations of washing tests^17,18^ both filtering the wastewater to quantify the amount of microfibres released. Different approaches were adopted to perform tests in washing machines. In particular, one garment/blanket^13,14,16^ or a 20 × 20 cm^2^ piece of garment^15^ was washed per time and, in some cases, for a short washing time (Hartline *et al*., 2016: 24–30 min^13^; Pirc *et al*., 2016: 15 min^14^). Filtration procedures also differ, but in general one type of filter was used (Pirc *et al*., 2016: 200 µm pore size^14^; Napper *et al*., 2016: 25 µm pore size^15^; Sillanpää *et al*., 2017: 0.7 µm pore size^16^) apart from Hartline *et al*.^13^, that used filters of two pore sizes (333 and 20 µm) but, as Sillanpää *et al*.^16^, filtered only an aliquot of wastewater.

Taking into account the state of the art, there is still a lack of knowledge on the effective contribution of the washing process of synthetic clothes to this environmental problem, on the real amount and on the dimensions of microfibres that are released in washing machine wastewater. In such scenario, this work aimed: (1) to obtain reliable quantitative data about the microplastic release from commercial synthethic clothes during washings in real household laundry machines; (2) to identify possible influences of textile characteristics on the release. For this purpose, wash trials at real scale were performed on commercial clothes by using a household washing machine. The whole wastewater volume was collected and filtered through subsequent filters with decreasing porosity. The analysis of the total volume of effluents allowed to recover more reliable data unaffected by errors induced by aliquot sampling. Moreover, the usage of a filtration process through decreasing porosity filters, assured the determination, besides the amount of microfibers released, also of their dimensions. Furthermore, the usage of a washing load compatible with the one generally used in domestic conditions and of a common washing program for “synthetics”, allowed to simulate as much as possible a real household washing process.

## Results

### Textile characteristics

**Figure 1:**
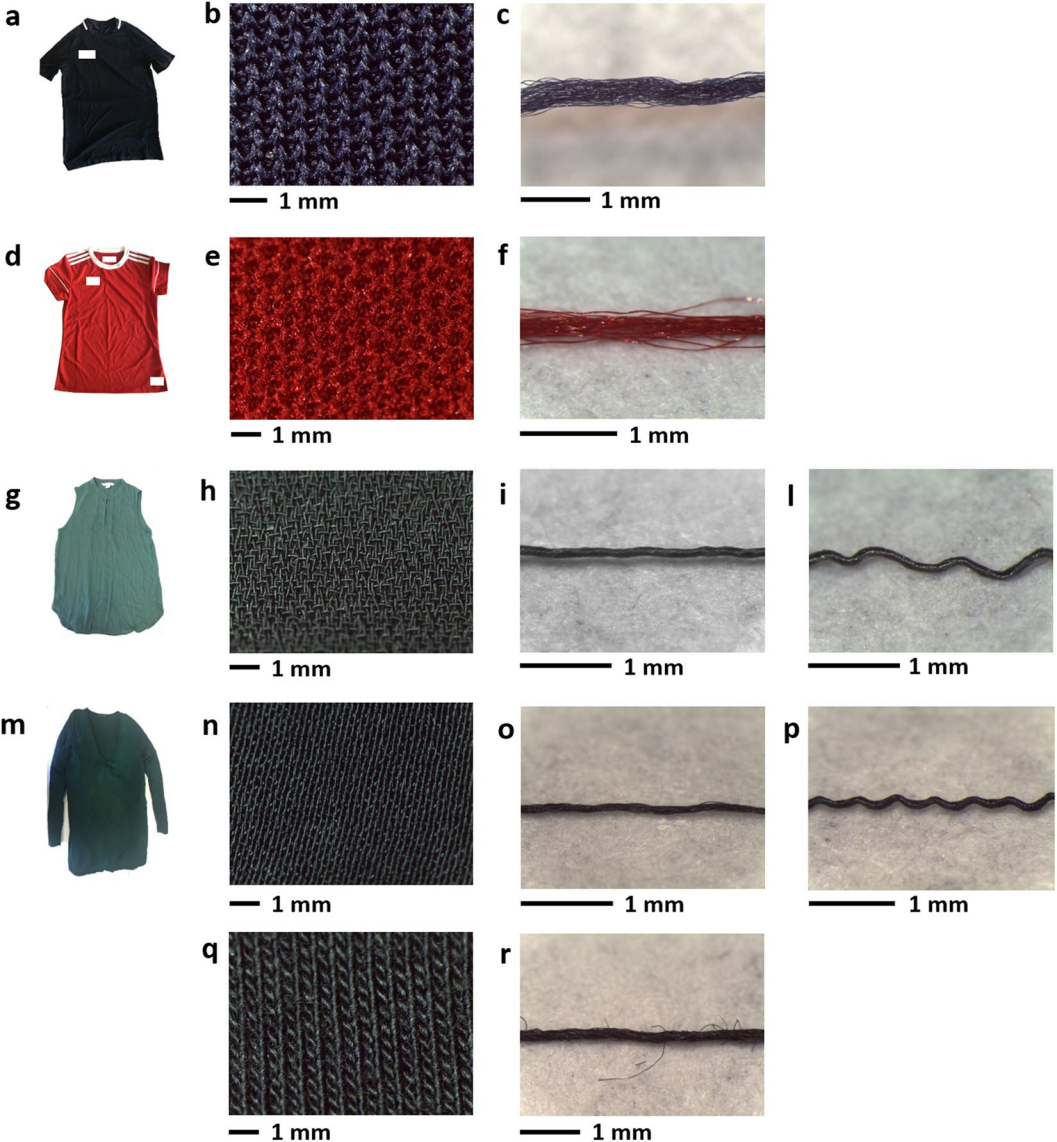
Pictures and optical micrographs of selected garments: (**a**) BT, a 100% polyester t-shirt, (**b**) plane surface and (**c**) yarn of BT; (**d**) RT, a 100% polyester t-shirt, (**e**) plane surface and (**f**) yarn of RT; (**g**) GB, 100% polyester blouse of which 65% is recycled polyester, (**h**) plane surface, (**i**) warp and (**l**) weft yarns of GB; (**m**) GT, a top whose front is made of 100% polyester and whose back is made of a blend of 50% cotton and 50% modal (**n**) plane surface, (**o**) warp and (**p**) weft yarns of GT front polyester part, (**q**) plane surface and (**r**) yarn of GT back modal/cotton part.

The analysis of the selected garments under optical microscope, whose micrographs are reported in Fig. 1, allowed to obtain information on their textile features. In general, textile fibres are spun into yarns, twisted in different way along fibre axis. The fibres constituting the yarns can be staple fibres, of comparatively short length, and filaments, which are fibres of indefinite length^19^. Yarns are mainly arranged in two structures: woven fabrics produced by interlacing two sets of yarns, the warp which runs in a lengthways direction and the weft which runs in a widthways direction and knitted fabrics produced by interlacing loops of yarn^20^. Moreover, the hairiness is defined as the presence of small fibres that protrude from the main yarn core^21^.

As possible to observe from Fig. 1b,c,e,f, both t-shirts made of 100% polyester, BT and RT, are knitted fabrics with low hairiness, whose yarn is made of continuous filaments with no twist. BT was made by weft knitting in a single jersey structure and RT was made by warp knitting. The 100% polyester blouse containing 65% of recycled polyester, GB, see Fig. 1h, has a satin weave structure with low hairiness. The two yarns constituting the woven are reported in Fig. 1i–l and they are both composed by filaments with the weft characterized by a slightly higher twist, 2510 t/m, than the warp, 2350 t/m. Finally, the top GT presents a double structure, observable in Fig. 1n,q. The front part of the top (Fig. 1n) is 100% polyester characterized by a satin weave structure, low hairiness and by both yarns made of continuous filaments (Fig. 1o,p), with a moderate twist in the case of the warp, 1274 t/m, and a higher one for the weft, 1669 t/m. The back of the top (Fig. 1q) is a blend of 50% cotton and 50% modal rib knitted with higher hairiness and the yarn is made of short staple fibres (Fig. 1r), very low twisted, 666 t/m.

### First washing cycle

**Figure 2:**
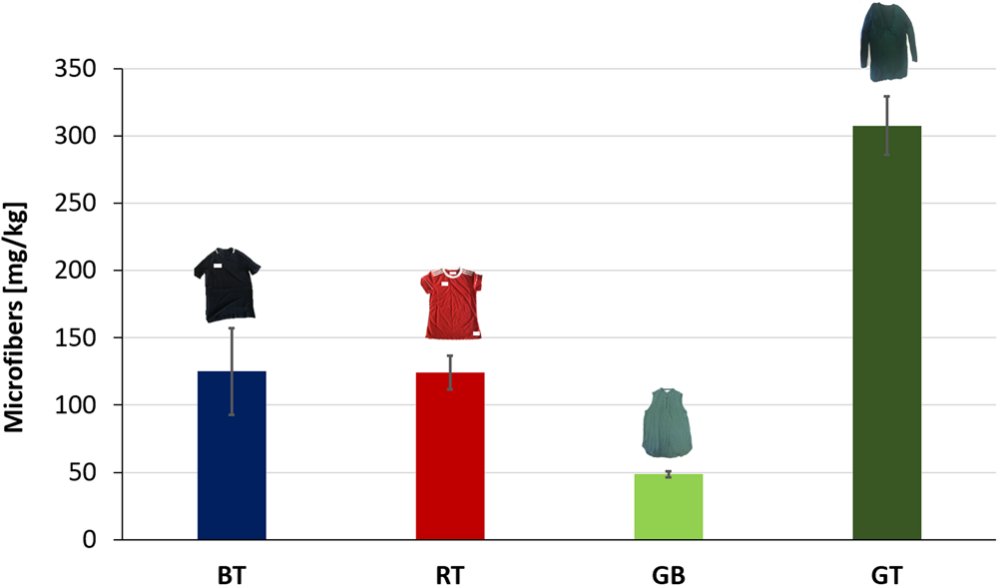
Microfibres released (expressed in mg/kg, M_a_ ± SD, n = 2) from BT, a 100% polyester t-shirt, RT a 100% polyester t-shirt, GB, a 100% polyester blouse of which 65% is recycled polyester, and GT, a top whose front is made of 100% polyester and whose back is made of a blend of 50% cotton and 50% modal.

The clothes were tested in washing trials to quantify the release of microfibres during washings. Each washing test was performed on a washing load of about 2–2.5 kg of identical clothes, with liquid detergent. The results of the microplastics released after the first washing for all garments are depicted in Fig. 2.

As possible to observe from Fig. 2, BT and RT released a comparable amount of microfibres during the washing, consisting of 125.0 ± 32.1 mg/kg and 124.1 ± 12.4 mg/kg of microfibres, respectively. This result can be explained considering that both t-shirts have the same fabric structure and yarn characteristics, so this similar behavior during washing tests is not surprising. Moreover, it indicates a high reproducibility into the amount of microfibres released by knitted polyester fabrics made with yarns constituted by continuous filaments. This last result also allows to demonstrate differences among the data notwithstanding the limitation in the replication of washing tests (n = 2) that inhibits statistical analysis. The large washing load tested, very close to real washing loads, implied the usage of a great amount of clothes per washing, that ranges from 16 to 22 garments depending on the washed clothes, complicating the number of replications affordable. Instead, GB released 48.6 ± 2.2 mg/kg of fibres, a value less than the half of that released from BT and RT. Such difference could be related to the fact that the yarns constituting GB have a higher twist compared to those of BT and RT, and are assembled into a woven structure, resulting in a more compact assembly that could make more difficult for fibres to slip from the fabric. The greatest amount of microfibres released came from GT, with a value of 307.6 ± 21.8 mg/kg. Such result is almost three times those obtained for BT and RT. GT has the most complex textile structure with a front polyester woven part and a back cotton/modal knitted part that could have different behaviors in the release. In order to allow more comparability among these results and the data reported in literature, the amount of microfibres was converted in number of microfibres, N. The results, in agreement with the gravimetric data, indicated that the greatest N was 1,500,000 microfibres, released by GT. 1,100,000 microfibres were released by BT and 770,000 by RT. The lowest number of microfibres, 640,000, was released by GB.

**Figure 3:**
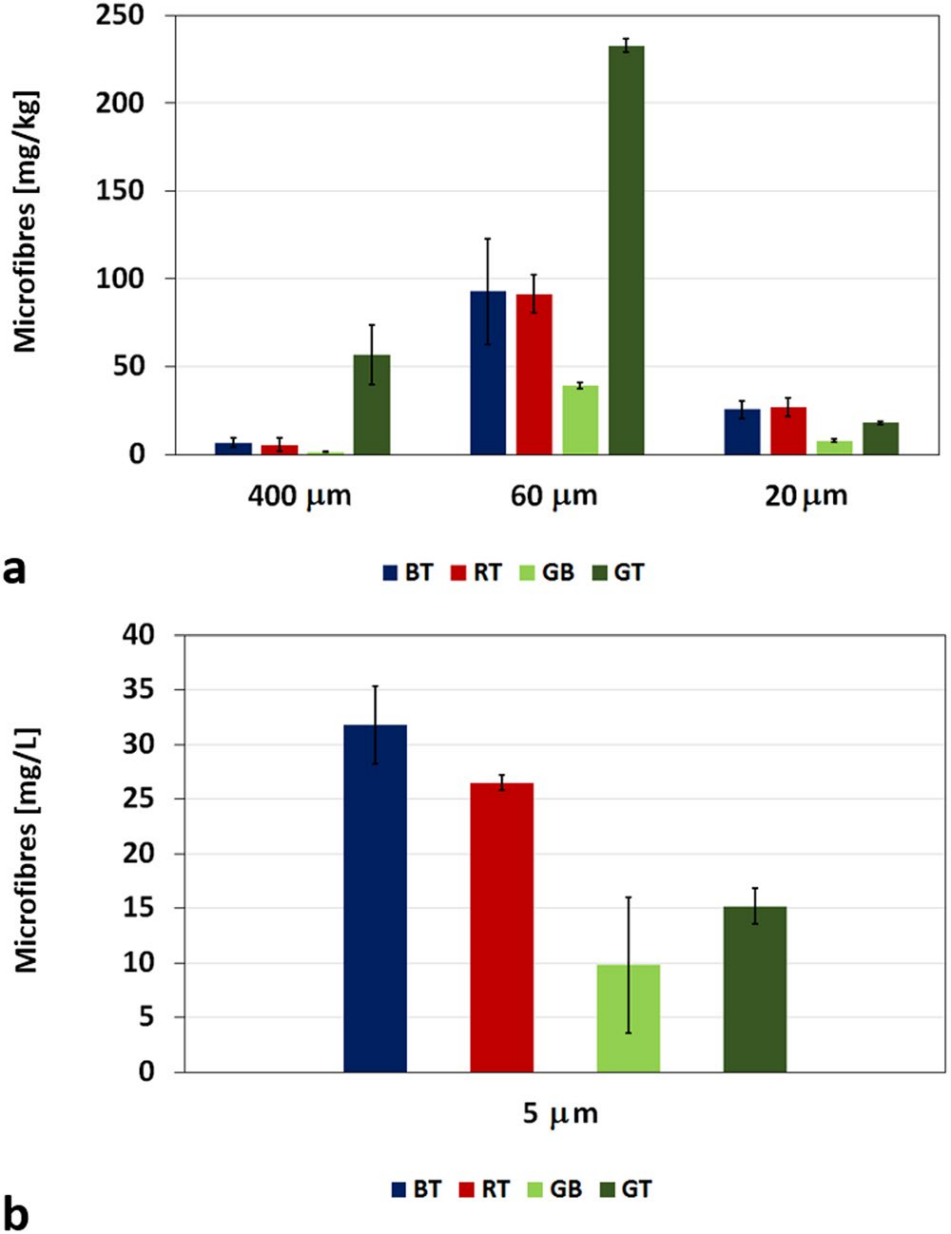
Microfibres recovered on: (**a**) 400 μm mesh, 60 and 20 µm pore size filters; (**b**) 5  µm pore size filters from the washing of BT, RT, GB, and GT.

The multistep filtration procedure allowed to obtain an indication of the dimensional ranges of released microfibres, since it was based on the recovery of microfibres on the 400 µm mesh, 60 µm and 20 µm pore size filters. In addition, 300 ml of wastewater were also filtered through a 5 µm pore size filter, obtaining an approximate concentration of the mg of microfibres per liter of water effluent; greater volumes were impossible to filter due to the clogging of the filter for its very small pore size. This last filtration provided evidence that small microfibres are present in the wastewater. Since the wastewater filtered on 5 μm pore size filter represented only a very small part of the total recovered wastewater, the results were not scaled to the entire effluent volume, since overestimation could be obtained with this calculation. The different amounts of microfibres recovered on each filter are reported in Fig. 3.

**Figure 4:**
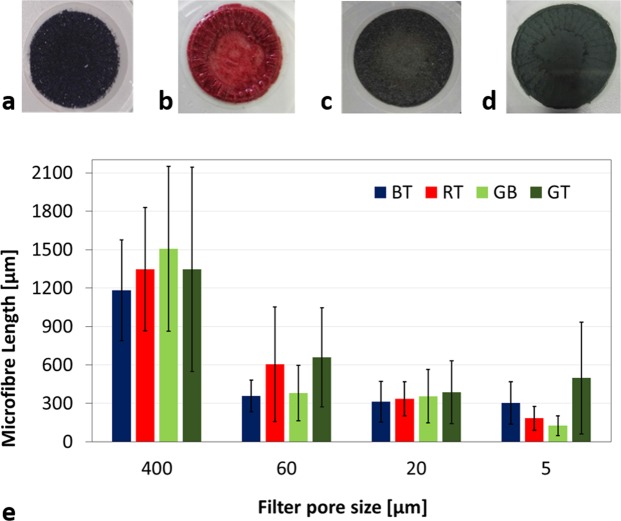
Pictures of the microfibres recovered on 60 µm pore size filters from the washing of (**a**) BT, (**b**) RT (**c**) GB, and (**d**) GT; (**e**) Length of microfibres released from BT, RT, GB and GT recovered on 400 μm mesh, 60, 20 and 5 μm pore size filters.

The greatest amount of microfibres recovered was the one collected on the filter of 60 µm pore size, pictures of the filter appearance after filtration are shown in Fig. 4a–d.

**Figure 5:**
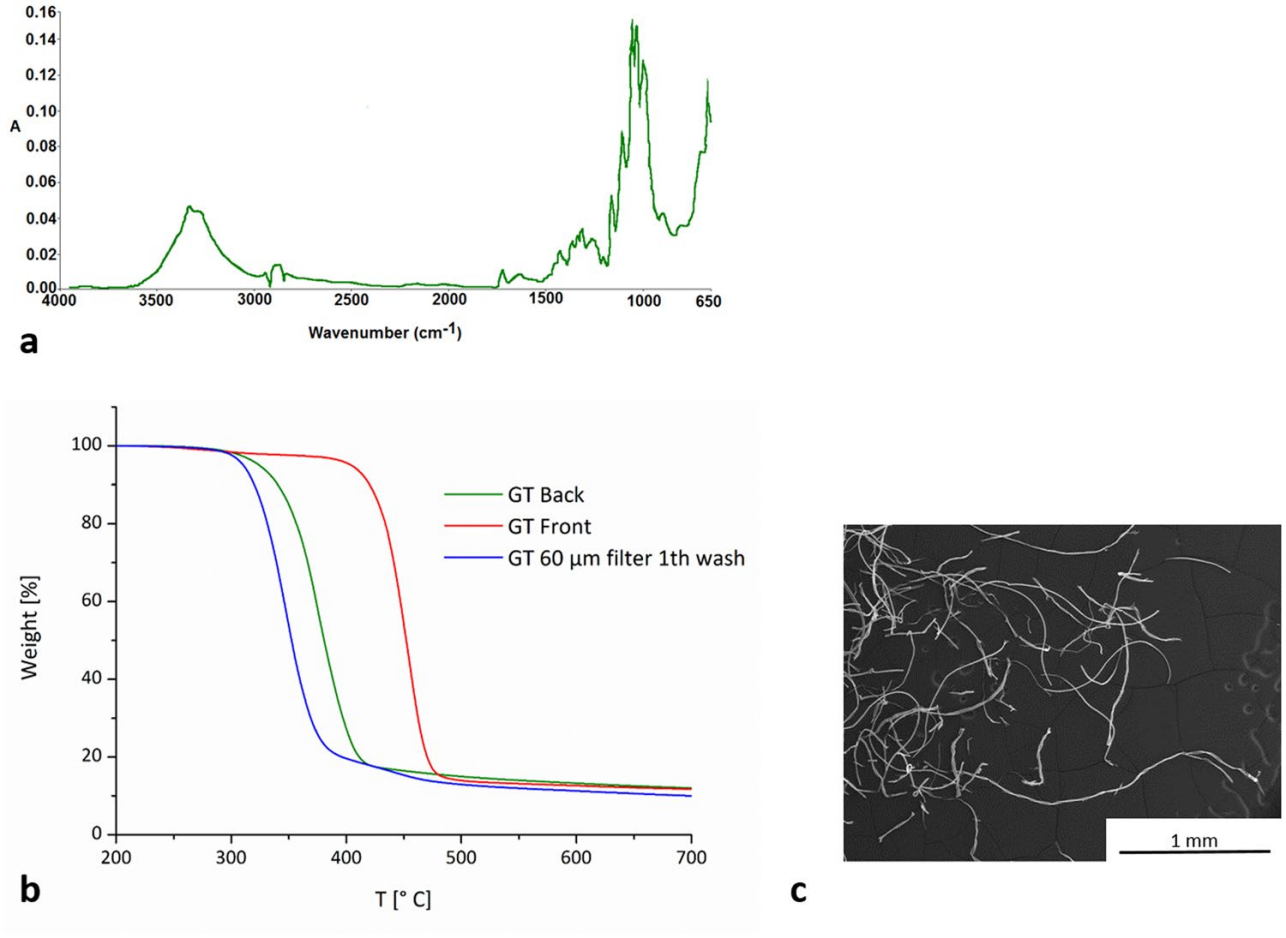
(**a**) FTIR spectrum of microfibres recovered from GT on the 60 μm pore size filter; (**b**) thermogravimetric curves of GT cotton/modal back part, polyester front part, microfibres recovered on 60  µm pore size filter; (**c**) SEM micrograph of GT microfibres representative of the fraction recovered on 60 µm pore size filter.

These findings indicate that most of the fibres that detach from the fabrics have dimensions compatible with such pore size. Of course, it should be considered that smaller fibres could be trapped inside this and other fractions. In order to evaluate the microfibre dimensions of the fractions recovered on the different filters, some amounts of microfibres for each filter were analyzed by optical or scanning electron microscopy, see Fig. S1 in the Supporting Information (SI). Figure 4e reported the average values of the length of the analysed microfibres, indicating a dimensional gradient that is function of the filter pore size. Microfibres with length ranging from 1180 to 1500 μm were blocked by the 400 μm mesh; those recovered on 60 and 20 μm pore size filters presented an average length of 360–660 μm and of 310–390 μm; finally, a range of 120–500 μm was stopped by 5 μm pore size filters. The diameter of the observed microfibres remained almost constant with the following values: 13.7 ± 1.8 μm for BT, 15.7 ± 3.3 μm for RT, 12.4 ± 1.8 μm for GB and 15.7 ± 4.9 μm for GT. For this last garment, it has to be highlighted that the dimensions of cotton microfibres presented a length similar to that of polyester, while its diameter was slightly greater (18.0 ± 2.1 μm for cotton, 13.3 ± 1.3 μm for polyester). The 400 µm mesh blocked similar amounts of fibres for BT and RT (6.7 and 5.6 mg/kg, respectively), very low for GB (1.4 mg/kg) and significantly higher for GT (56.8 mg/kg). The same trend GT >> BT,RT >> GB, was observed also for the fibres recovered on the 60 µm filter, whereas fibres collected on 20 and 5 µm filters showed a different behavior. In fact, for both of them, the fibres released from BT and RT were of similar amounts (20 µm: 25.5 mg/kg for BT, 27.0 mg/kg for RT; 5 µm: 31.8 mg/L for BT, 26.5 mg/L for RT) but slightly greater than the amount released from GT (20 µm: 18.0 mg/kg; 5 µm: 15.2 mg/kg). For all garments the 60 µm filter was able to retain around 75–80% of the total amount of microfibres released per wash. For BT, RT and GB, 400 and 20 µm filters retained around 5% and 20% of the total release but, in the case of GT, such values were reversed (400 µm: around 20%; 20 µm: around 5%). The nature of the microfibres recovered on 60 μm pore size filters, that represented the most abundant microfibre fraction, was confirmed by using FTIR spectroscopy. The FTIR spectra of BT, RT and GB confirmed the polyester composition of the recovered microfibres and are reported in Fig. S2 in the SI. The spectrum of the microfibres released from GT, reported in Fig. 5a, is typical of cellulosic fibres. The broad band in the region 3700–3000 cm^−1^ is due to the OH-stretching vibrations containing the contribution of both hydroxyl groups interacting via intra-molecular hydrogen bonding centered at around 3340 cm^−1^, and via inter-molecular hydrogen bonding centered at around 3280 cm^−1^. The bands at 2940 and 2880 cm^−1^, are assigned to CH_2_ asymmetrical and symmetrical stretching. The bands observed at 1720 and 1640 cm^−1^ are attributed to CO stretching and to OH bending of adsorbed water, respectively. The absorption bands at 1429, 1369, 1312 and 1204 cm^−1^ are due to OH in-plane deformation, CH bending, CH_2_ rocking and to the CO stretching mode of the pyranose ring. The 1161 cm^−1^ vibration is attributed to anti-symmetrical bridge COC stretching within cellulose. The vibrations at 1003 and 986 cm^−1^ are attributed to C-O and ring stretching modes, and the 897 cm^−1^ vibration is assigned to the β-linkage of cellulose^22^. In addition, thermogravimetrical analysis was performed on GT microfibres. Figure 5b reports the thermogravimetric curves of GT cotton/modal back part, GT polyester front part, and of GT microfibres recovered on 60 µm pore size filter.

The GT front presents a single step thermal degradation, with a temperature of max weight loss (T_max_) of 454 °C, due to the decomposition of the main chain of polyester^23^. The GT back part has also a single step degradation but shifted to lower temperatures with a T_max_ at 377 °C, ascribable at cellulosic degradation of the blend cotton/modal. It is reported in literature that cotton and viscose have a similar thermal degradation behavior, with the degradation of viscose starting at lower temperatures compared to cotton^24,25^. However, in the analysed sample of GT back, no difference in the degradation of both materials was detected. Instead, the microfibres recovered on the 60 µm filter present a two-step thermal degradation: the first start at 200 °C and has a weight loss of around 81% (T_max_ = 348 °C), the second starts at 406 °C and loose around 9% of weight (T_max_ = 450 °C). Comparing such results with the thermal degradations of GT front and back parts, it appears clear that the first step could be attributed to the degradation of the cotton/modal microfibres, whereas the second step is ascribable to the degradation of polyester. Then, around the 80% of the amount of microfibres released from GT during washing are of cellulosic nature, released from the back part. Moreover, the presence of cellulosic microfibres on the 60 μm pore size filter was also evaluated by SEM analysis as represented in Fig. 5c.

### Subsequent washing cycles

**Figure 6:**
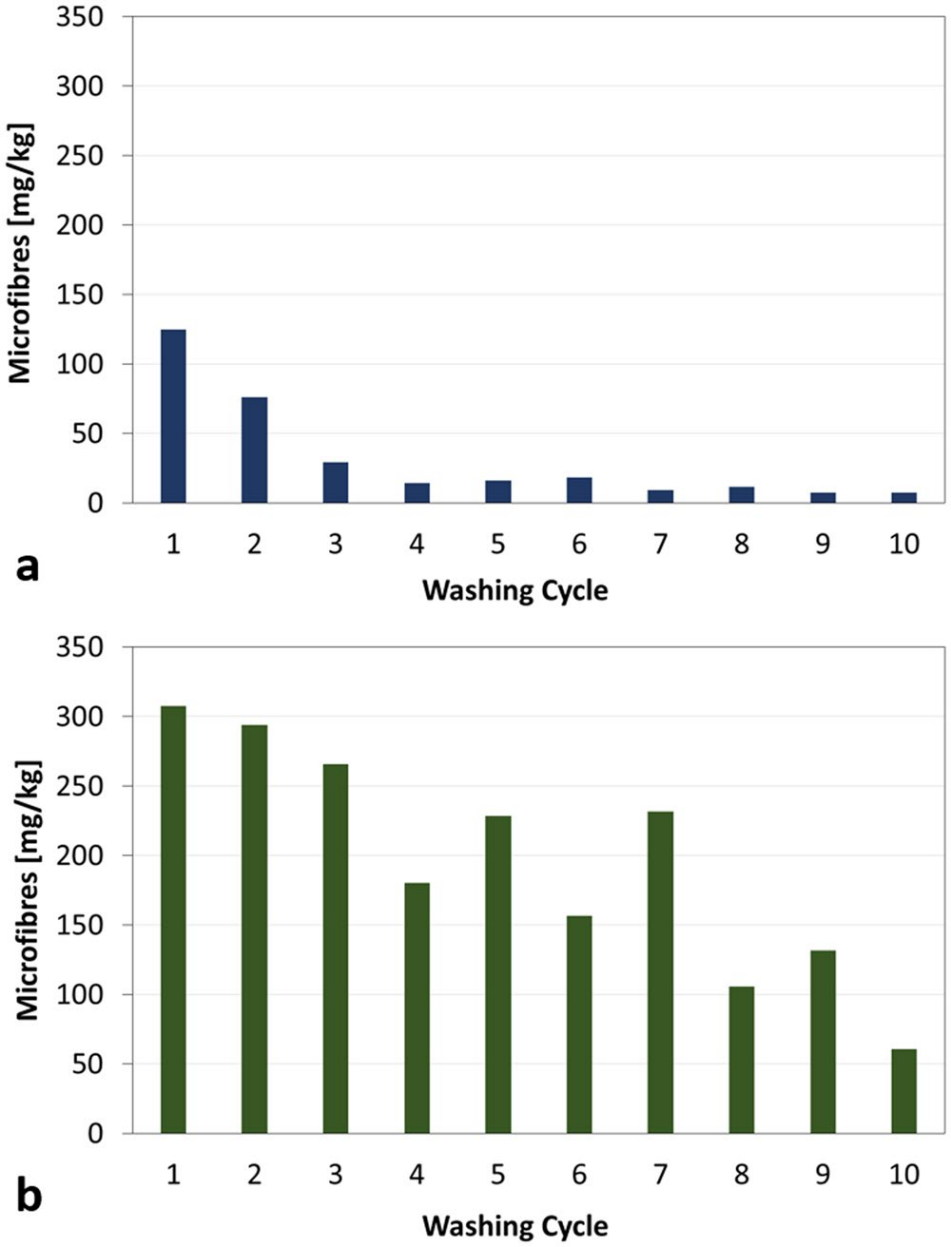
Total amount of microfibres released during 10 washing cycles from: (**a**) BT and (**b**) GT.

**Figure 7:**
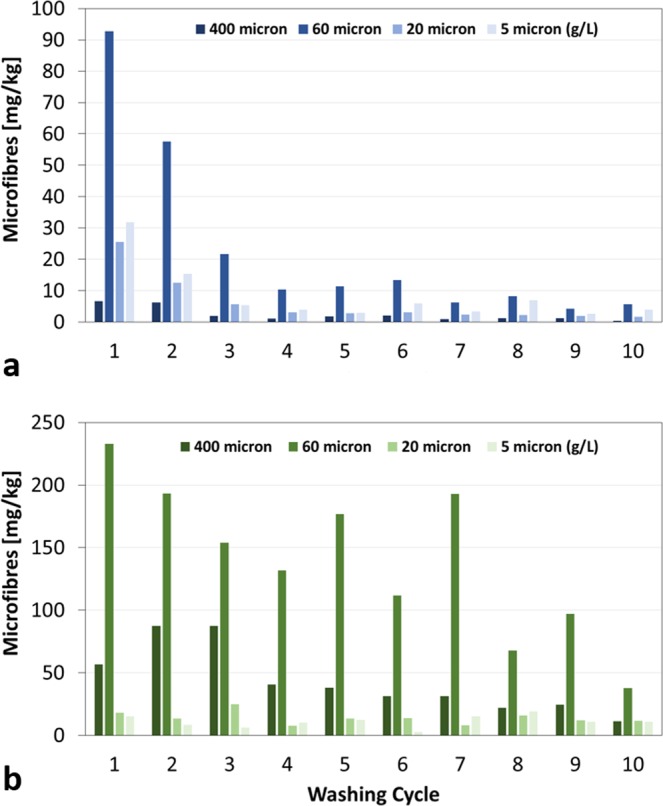
Microfibres recovered on 400 μm mesh, 60 and 20 µm pore size filters (expressed in mg/kg), and on 5 µm pore size filters (expressed in mg/L), during 10 washing cycles from: (**a**) BT and (**b**) GT.

Due to their completely different behavior, BT and GT were selected to undergo up to 10 washing cycles. The morphological analysis of BT and GT surfaces before and after 10 washing cycles indicated that no visible damages of the fabrics occurred during washings. SEM micrographs of BT and GT clothes before and after the washings are reported in Fig. S3 in the SI. Figures 6 and 7 summarize the results of this investigation. After 4^th^–5^th^ cycles, the total amount of microfibres released from BT reached a plateau, Fig. 6a, on the contrary, the release from GT showed a slightly decrease after 4^th^–5^th^ cycles but no plateau was reached up to the 10^th^ cycle, Fig. 6b. The same trend was observed in the microfibres recovered on 60 µm pore size filters, Fig. 7. The amount of microfibres released from BT recovered on 60 µm pore size filters, Fig. 7a, decreased until the 4^th^ cycle, while that released form GT and recovered on 60 μm pore size filters slightly decreased until the 4^th^ cycle and then presented an oscillating pattern, Fig. 7b.

**Figure 8:**
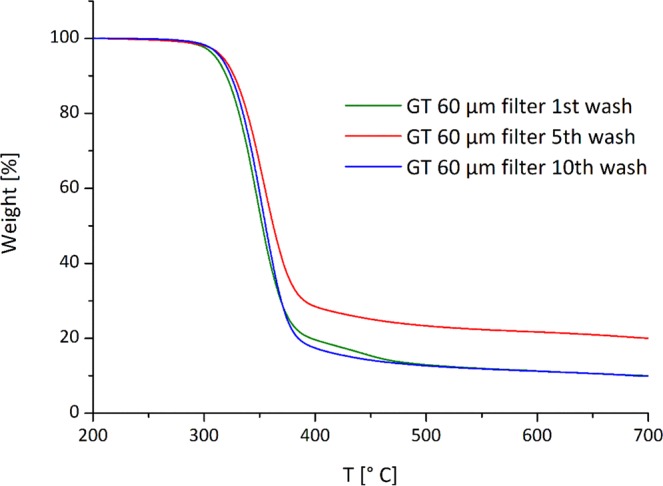
TGA curves of the microfibres recovered from 60 µm pore size filters of 1^st^, 5^th^ and 10^th^ washes performed on GT.

Also in this case, a thermogravimetric analysis was performed on microfibres recovered on 60 µm pore size filters after the 5^th^ and 10^th^ washing cycles and compared with the results obtained from the 1^st^ washing cycle. The TGA curves reported in Fig. 8, showed that compared to the thermal degradation of the fibres from the 1^st^ wash, both microfibres from the 5^th^ and 10^th^ washes have a single step degradation, with a close T_max_ (353 °C for the 5^th^, 354 for 10^th^). This result seems to indicate that the microfibres released during the 5^th^ and 10^th^ washes were mainly released from the cotton/modal back part.

## Discussion

The effective contribution of the washing process of synthetic clothes to the environmental problem caused by microplastic pollution was assessed in this work. The analytical procedure reported introduces three important simultaneous novelties: (1) real washing load and program were tested; (2) all the wastewater coming from the washing machine was analysed; (3) different pore size filters were used in a multistep filtration procedure. These allowed to obtain reliable quantitative data about the amount, dimensions and nature of microplastics released during washing. The microfibres released ranged from 124 to 308 mg for kg of washed fabric depending on the washed garment, that indicate a release of 640,000–1,500,000 microfibers.

A comparison of the release of microfibres herein reported with data obtained from other studies, is not immediate due to the different washing conditions and quantification methods used. However, the release of BT and RT (125.0 ± 32.1 mg/kg and 124.1 ± 12.4 mg/kg, respectively) were much lower than those reported in a previous work by Pirc *et al*.^14^, that tested polyester fleece blankets whose multifilament structure can release microfibres more easily. Sillampää *et al*.^16^ reported a range of microfibres released from cotton and polyester clothes that is 0.12–0.33% w/w, which is instead much greater than the amounts reported in the present work, even considering GT.

The decreasing trend in microplastic release after consecutive washings recorded for BT, seems to be in line with results from other works^14–16^. Concerning the type of textile, the greatest amounts released by GT, a blend of polyester/cotton/modal, is in contrast with the low quantities recorded for other cotton/polyester blend shirts^15^. Nevertheless, the observations on the yarn characteristics are in agreement with the work of Carney Almroth *et al*.^17^, concluding that yarns made of filament fibres with high twist could contribute to the reduction of microplastics released during washing processes of synthetic clothes. In fact, comparing the textile characteristics of the tested fabrics, GB released less than BT and RT, the two t-shits with a similar structure (knitted, continuous filaments, no twist, low hairiness), because it is made of a woven structure with twisted yarns. The difference in the textile structure among GB, BT and RT does not allow to ascertain if the different polyester composition among GB (made of 65% recycled polyester) and BT and RT (made of 100% polyester) could have a role in the microfibre release. The highest release of microfibres was obtained during washing of GT, a top that presents a polyester part made by continuous filaments but with a lower twist than GB, and a cellulosic part made by short staple fibers with a very low twist. In this last case, additional analyses indicated that about the 80% of the released microfibres were of cellulose, suggesting that a strong influence in the release may be due to the short staple fibers which could more easily be released from the fabric compared to the continuous filaments more twisted of the woven structure of the front. In fact, as reported in literature, in natural fibres staple length is not well defined and every batch consists of fibres varying in length over a wide range^26^. Such results allowed to identify some parameters that could be related to the release of microfibres. In particular, clothes with yarns made of continuous filaments, high twist and low hairiness released less microfibres during washing. These results could indicate possible changes in textile design for the apparel industries, which could contribute to the reduction of microplastic release. Nevertheless, considering that microfibres are released either from fabrics with yarns made of short staple fibers, either from fabrics containing yarns made with continuous filaments, it should be difficult for the moment to define a threshold fibre length value below which there is microfibre release and above which there is no release.

Regarding the dimensions of the released microfibres, the fact that the most abundant fraction of microfibres was recovered on a filter of 60 µm pore size and was characterized by a length of 360–660 μm, indicates that the dimensions of these microfibres are compatible with those of microplastics found in water effluents (>300 μm) coming from WWTPs^6,8,9^, and of microfibres (>50 μm) found in marine sediments and ingested by fauna^10–12^.

The experimental part on subsequent washing tests pointed out that while 100% polyester clothes reach a plateau after 4–5 washing cycles, the release of microfibres from garments with a mixed composition of polyester/cotton/modal is still very strong after 10 washing cycles and is mainly composed by cellulosic microfibres. The fate of cellulosic fibres in marine environment is still not clear but their presence have been reported in freshwater and atmospheric environments^27^ and more research on their possible impact is needed. In conclusions, this work highlighted the necessity to mitigate microfibre release from synthetic clothes acting directly on fabric structure. Solutions involving surface treatments of synthetic fabrics have already been suggested showing promising results in term of microfibre reduction^28,29^. Nevertheless, actions should be undertaken also at textile design stage in order to avoid the usage of textile characteristics that increase the release.

## Methods

Four types of commercial garments were kindly supplied in more than one item by Plastic Soup Foundation (Amsterdam, the Netherlands): a blue t-shirt (100% polyester, code BT, Fig. 1a), a red t-shirt (100% polyester, code RT, Fig. 1d), a green sleeveless blouse (100% polyester of which 65% is recycled polyester, code GB, Fig. 1g), a green long sleeved top (the front is made of 100% polyester and the back is made of a blend of 50% cotton and 50% modal, code GT, Fig. 1m). The identity of each fabric type was confirmed by Fourier Transform Infrared (FTIR) spectroscopy. The fabrics were observed using a LEICA M205C light microscope to assess textile characteristics. The textile characteristics of the fabrics, yarns and fibres constituting the garments were determined. The construction of the fabrics constituting the garments was determined analyzing optical micrographs of the fabric surfaces. The yarn twist in turn/meter, was measured by using Eq. 1

1 $$T=\frac{\tan \,{\rm{\theta }}\,}{{\rm{\pi }}{\rm{d}}}$$

where θ is the angle formed by the fibre in the yarn with the yarn axis, and d is the diameter in meters^30^.

The commercial liquid laundry detergent used in the washing tests for the release has the following composition: 5–15% of anionic and non ionic surfactants; <5% of soap and phosphonates; optical whitening agents; enzymes and perfume.

Cotton lab coats and nitrile gloves were worn during all the experimental work.

Washing tests were performed using a Bosch washing machine serie 4 varioperfect WLG24225i, at 40 °C, for 107 min and 1200 rpm, the washing conditions of the program for synthetics. The commercial liquid detergent was used in the dose recommended by the supplier. Each washing test was performed on new garments. More items of the same type of garment were washed together in order to reach a washing load of 2–2.5 kg. Two replicates of each wash tests were performed. Ten consecutive washing cycles were performed on BT and GB in order to evaluate the microfibre release vs washing cycle. The garments were dried at air between the cycles, to simulate real laundry habits. Cross-contamination of fibres between washes was prevented by running two consecutive empty washing cycles, the first at 60 °C, 1200 rpm, 135 min, the second at 40 °C, 1200 rpm for 30 min. 4 blank tests were performed to assess the level of contaminations between sequential washes. No microfibres were detected on the filters in the blank tests.

The analytical procedure adopted for the evaluation of microfibre release, consisted in the filtration of the wastewater coming directly from the drainpipe of the washing machine, with a 400 µm pore size mesh. Then, the wastewater was recovered in tanks and filtered by means of a peristaltic pump (SP 311/60 Velp Scientifica) connected with Tygon tubes, throughout a nylon net filter with a 60 µm pore size (Merck Millipore) and then through a nylon net filter with a 20 µm pore size (Merck millipore). Finally, 300 ml of the filtered wastewater were additionally filtered through a PVDF membrane of 5 µm pore size (Durapore®, Merck Millipore). A drawing of the filtration system is reported in Fig. S4 in the SI. When clogging of the filters occurred, the filtration was stopped, clogged filters were removed and stored, new filters were applied and the filtration restarted. At the end of each tank, 1 L of distilled water was poured into, the tank was shaken, and the water filtered. Such procedure was carried out twice for each tank to collect possible fibres that remained attached to the surface of the tanks. Finally, 1 L of distilled water at 70 °C was fluxed in the filtration system to clean the filters from excess of detergent. All filters were dried in oven at 105° for 1 h and then weighted. The filters were weighted before and after the filtration in order to evaluate the amount in grams of microfibres released, that was normalized for the washing load. For each washing test, two replicates were performed and the amount of microfibres released was calculated as average value (M_a_), along with the standard deviation (SD). To avoid cross contamination of fibres among the different filtrations, tygon tubes, filter holders and tanks were cleaned with distilled water and with a jet of compressed air. The rinsed tanks were not tested for residual fibres but it should be underlined that the usage of a peristaltic pump allows to avoid any residual water in the tanks and two rinsing were considered sufficient to avoid microfibre loss or better to avoid mistakes in the amount of microfibres released.

The average values of the length, L, and diameter, D, of the released microfibres were evaluated along with the standard deviation, through the measurements of 40 microfibres per wash by using Image J (release 1.43 u). For this purpose, microfibres recovered on 5, 20 and 60 μm pore size filters were analysed using a Scanning Electron Microscope (SEM) Quanta 200 FEG (FEI, The Netherlands). SEM observations were performed in low vacuum mode (PH2O = 0.7 torr), using a large field detector (LFD) and an accelerating voltage of 30 kV. Microfibres recovered on the 400 μm pore size mesh were analysed by using a LEICA M205C light microscope.

The amount of microfibres was converted in number of microfibres, N, assuming that the fibres were all of cylindrical shape and using Eq. (2):

2 $$N=\frac{\frac{{M}_{tot}}{\rho }}{\pi \frac{{D}^{2}}{4}L}$$

where M_tot_ is the weight of microfibre released during the washing, ρ is the density of the material, L is the mean microfibre length and D is the mean microfibre diameter^15^.

The chemical composition of the microfibres recovered was confirmed by using a Fourier Transform Infrared (FTIR) spectroscopy. Spectra were acquired with a Perkin Elmer Spectrum One FTIR spectrometer, equipped with the Universal ATR accessory, using 16 scans and a resolution of 4 cm^−1^, over the range 4000–650 cm^−1^.

To assess the nature and relative amount of microfibres released during the washing of GT, whose front is made of polyester and the back of a cotton and modal blend, a thermogravimetric analysis was performed on about 5 mg of microfibres recovered on the filter with 60 µm pore size as well as on neat samples, about 5 mg, cut from the front and the back of the top. Samples were placed in an open platinum pan and heated from 30 to 800 °C at the rate of 10 °C min^−1^ under nitrogen atmosphere (flow rate: 40 mL min^−1^) in a Pyris 1 TGA from Perkin–Elmer (Waltham, MA, USA).

## Supplementary information

Supporting Information
