# Supplementary material for: The contribution of washing processes of synthetic clothes to microplastic pollution
Source: Sci Rep. 2019 Apr 29;9:6633. doi: 10.1038/s41598-019-43023-x (PMC6488573; doi:10.1038/s41598-019-43023-x)
Supplement: Supplementary file 1 — Supporting Information [file 41598_2019_43023_MOESM1_ESM.pdf]

The contribution of washing processes of synthetic clothes to microplastic pollution

Francesca De Falco[1], Emilia Di Pace[1], Mariacristina Cocca[1*], Maurizio Avella[1]

1 Institute for Polymers, Composites and Biomaterials, National Research Council of Italy, Via Campi Flegrei, 34 - 80078 Pozzuoli (NA), Italy

Corresponding Author:

* Mariacristina Cocca

Institute for Polymers, Composites and Biomaterials, National Research Council of Italy, Via Campi Flegrei, 34 - 80078 Pozzuoli (NA), Italy

tel. +390818675363

e-mail address mariacristina.cocca@ipcb.cnr.it

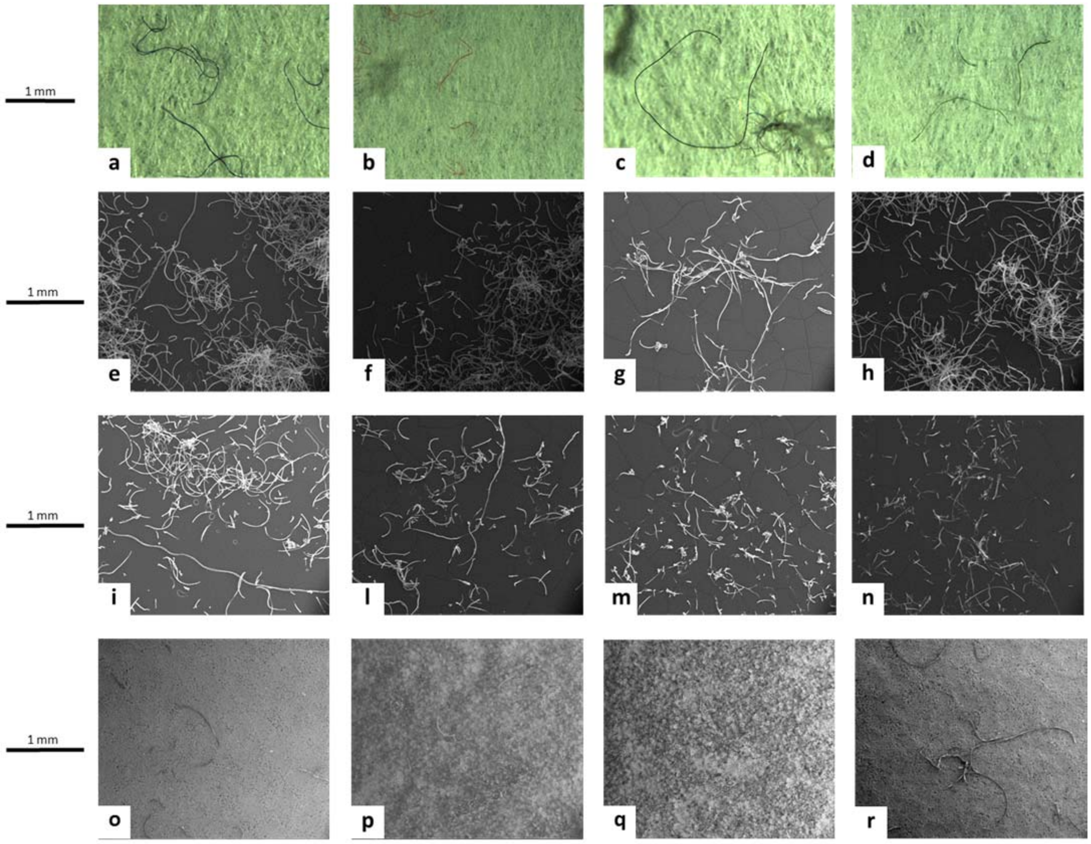

Figure S1. Optical micrographs of microfibers sampled by 400 μm pore size mesh released by a) BT, b) RT, c) GB, d) GT; SEM micrographs of microfibers sampled by 60 μm pore size filters released by e) BT, f) RT, g) GB, h) GT; SEM micrographs of microfibers sampled by 20 μm pore size filters released by i) BT, l) RT, m) GB, n) GT; SEM micrographs of microfibers sampled by 60 μm pore size filters released by o) BT, p) RT, q) GB, r) GT.

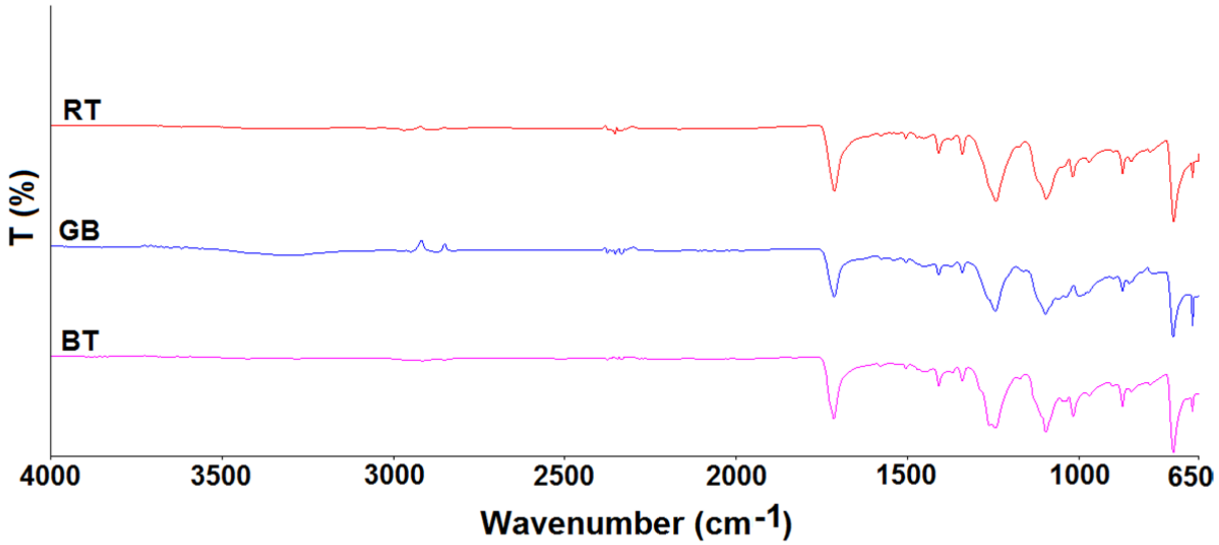

Figure S2. FTIR spectra of RT, GB and BT

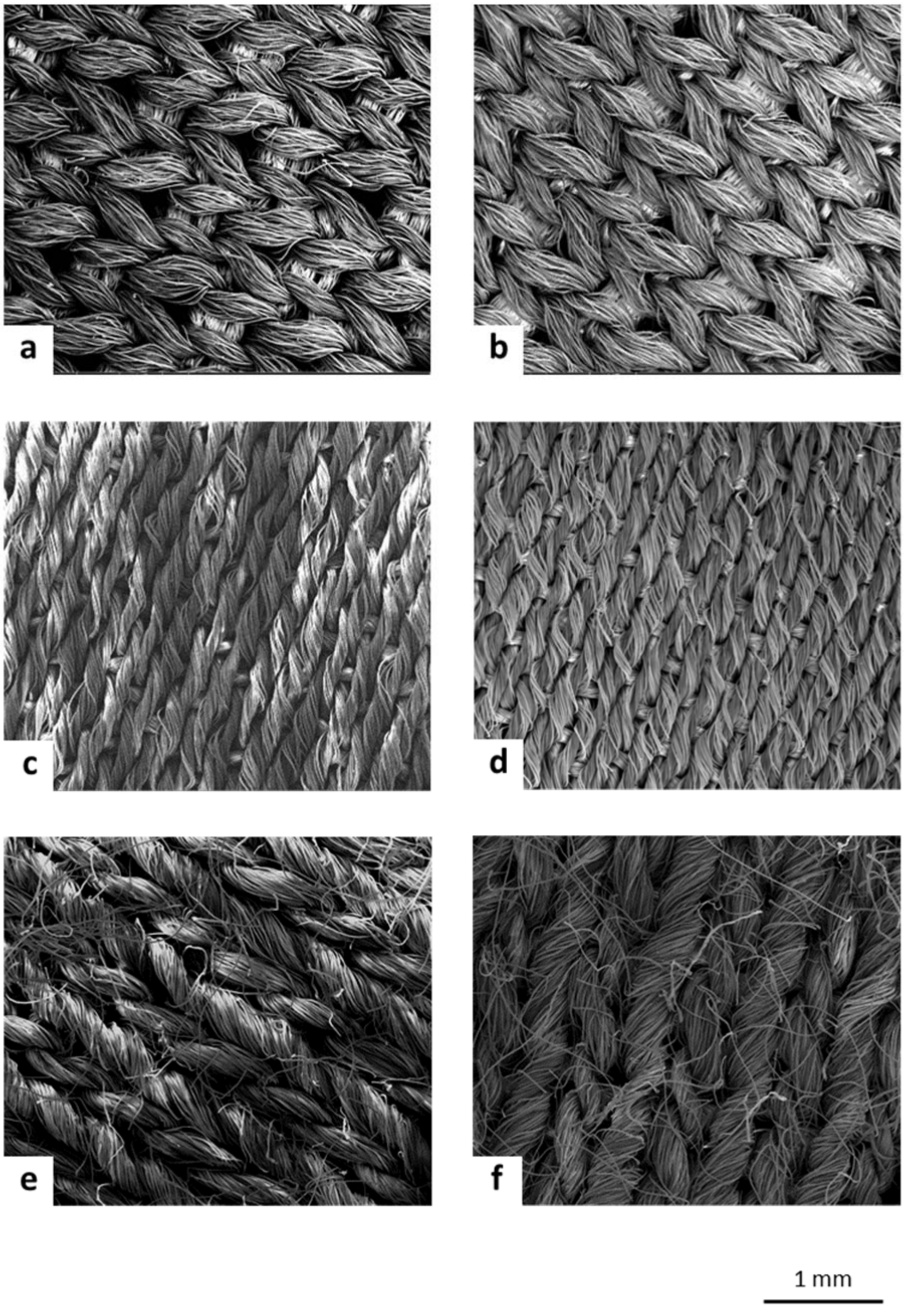

Figure S3. SEM micrographs of a) BT before washing test, b) BT after 10 washing cycles, c) GT polyester front part before washing, d) GT polyester front part and after 10 washing cycles, e) GT cotton/modal back part before washing, f) GT cotton/modal back part after 10 washing cycles.

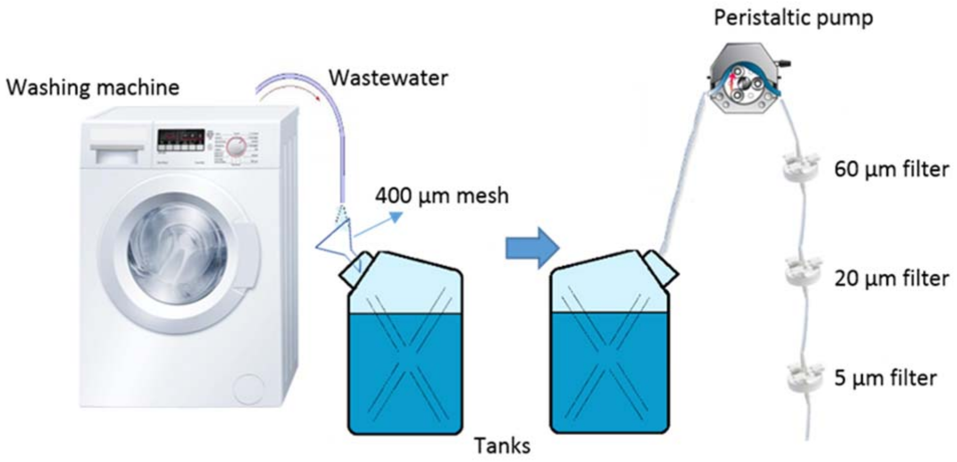

Figure S4. Drawing of the filtration system
